# Roxadustat in Kidney Transplant Recipients with ESA-Hyporesponsive Anemia: A Prospective Single-Center Cohort Study

**DOI:** 10.3390/life16050815

**Published:** 2026-05-13

**Authors:** Antonio Franco, Patricio Más-Serrano, Iván Beltrá-Picó, Elena de la Cruz, Noelia Balibrea, Nuria Bondia, Javier Perez-Contreras

**Affiliations:** 1 Department of Nephrology, University General Hospital Dr. Balmis of Alicante, 03010 Alicante, Spain; eledelacruz04@gmail.com (E.d.l.C.); nobala@gmail.com (N.B.); nuriab11294@gmail.com (N.B.); perez_fra@gva.es (J.P.-C.); 2 Alicante Institute for Health and Biomedical Research (ISABIAL-FISABIO Foundation), 03010 Alicante, Spain; mas_pat@gva.es (P.M.-S.); ivanbeltrap@gmail.com (I.B.-P.); 3 Pharmacy Department, University General Hospital Dr. Balmis of Alicante, 03010 Alicante, Spain

**Keywords:** roxadustat, refractory anemia, renal transplant

## Abstract

Roxadustat, an oral hypoxia-inducible factor prolyl hydroxylase inhibitor, stimulates erythropoiesis. This prospective single-center, non-randomized, single-arm cohort study enrolled renal transplant recipients with refractory anemia, defined as hemoglobin (Hb) ≤10 g/dL despite receiving the maximum doses of erythropoiesis-stimulating agents for 12 weeks. The studied parameters were Hb, ferritin, iron saturation index, glomerular filtration rate (eGFR), and C-reactive protein (CRP). Follow-up assessments were conducted at 4, 8, and 12 weeks after starting Roxadustat. The primary endpoint was the proportion of patients achieving Hb > 11 g/dL at 12 weeks. Secondary endpoints included changes from baseline in studied parameters and adverse effects. Twenty recipients (11 male, 9 female) with a median age of 69.0 years and a median time post-transplant of 62.5 months were included. Median baseline eGFR was 16.5 mL/min/1.73 m^2^. At 12 weeks, 19 of 20 (95%) achieved Hb > 11 g/dL. Median Hb increased significantly from 9.1 g/dL to 11.5 g/dL, with a median individual change of +2.7 g/dL (IQR 1.7–3.4; *p* < 0.001). The only non-responder increased Hb from 9.5 to 10.2 g/dL. Ferritin decreased significantly over 12 weeks, whereas no statistically significant changes were observed in transferrin saturation, CRP, or eGFR. No serious adverse events were observed. In this prospective cohort, roxadustat was associated with short-term hemoglobin improvement and high Hb target attainment; however, these findings should be interpreted cautiously given the single-arm design and limited sample size.

## 1. Introduction

Anemia is one of the most frequent and clinically relevant complications in patients with chronic kidney disease (CKD). Its prevalence rises as renal function declines, and this progression is not merely a laboratory phenomenon: it has direct consequences for physical performance, cardiovascular risk, hospitalization, and quality of life. In advanced CKD, anemia is observed in more than 50% of patients at stage 4 and in over 90% of those at stage 5 who require dialysis [1]. These data show that anemia should not be considered an isolated associated finding, but rather a core component of the CKD syndrome.

Kidney transplant recipients are not exempt from this problem. Although transplantation can improve kidney function and reduce many uremia-related abnormalities, post-transplant anemia remains common, with prevalence estimates ranging from 20% to 51% depending on population characteristics, follow-up periods, and diagnostic criteria [2,3,4]. In this setting, anemia often has a multifactorial basis and may persist despite optimization of standard management.

The pathophysiology of anemia in CKD and after kidney transplantation is complex. Erythropoietin deficiency is usually the dominant mechanism, but iron deficiency (absolute or functional), inflammation, reduced graft function, intercurrent disease, and medication-related bone marrow effects can all contribute [1,3,4,5]. In routine nephrology practice, treatment with iron therapy and erythropoiesis stimulating agents (ESAs) corrects anemia in a large proportion of patients [6]. Nevertheless, a subset remains suboptimally controlled despite high ESA exposure. Hyporesponsiveness to erythropoietic therapy may be potentiated by cytokines such as TNF-alfa, and this might then antagonise the anti-apoptotic action of erythropoietin on erythroid progenitor cells, thus reducing responsiveness to exogenous erythropoietic therapy [7].

This difficult subgroup is commonly described as having refractory or hyporesponsive anemia. According to National Institute for Health and Care Excellence (NICE) criteria, this situation is defined by failure to reach target hemoglobin despite high ESA doses (around 300 IU/kg/week), or by the need for persistently very high doses to maintain target values [8]. Refractory anemia is associated with higher morbidity and mortality [9], and its diagnosis requires careful exclusion of additional causes of poor response, including occult blood loss, intercurrent inflammatory or infectious disease, pure red cell aplasia, aluminum toxicity, and other hematologic conditions [10].

In recent years, hypoxia-inducible factor prolyl hydroxylase inhibitors (HIF-PHIs) have emerged as an alternative strategy for anemia management. Roxadustat, a reversible HIF-PHI, activates adaptive pathways that increase endogenous erythropoietin production within physiologic ranges, improve iron mobilization and transport, and reduce hepcidin levels, particularly in inflammatory contexts [11]. This broader mechanism is relevant because many refractory cases are characterized by chronic inflammation [12] and functional iron restriction [10], where traditional approaches may lose effectiveness.

Kidney transplant recipients may be especially suitable candidates for this therapeutic concept. Chronic low-grade inflammation is common in this population [12], and functional iron deficiency is frequently observed even when ferritin stores appear adequate (for example, ferritin > 100 µg/L with transferrin saturation < 20%) [10]. Therefore, a therapy that supports erythropoiesis while improving iron bioavailability could provide benefit where conventional ESA-centered protocols are insufficient.

From a clinical perspective, treating anemia in transplant recipients is not just a matter of correcting hemoglobin values. Post-transplant anemia has been associated with reduced quality of life, increased cardiovascular events, and poorer graft and patient survival [2,13,14]. A treatment that safely improves anemia in this context may therefore have broader prognostic implications beyond short-term hematologic correction.

Evidence for roxadustat in kidney transplant recipients is still limited. Available studies are few, conducted mainly in Asian populations, and generally focused on post-transplant anemia in broader terms rather than strict refractory anemia cohorts [15,16,17,18,19,20,21]. As a result, uncertainty remains regarding the magnitude of benefit and safety profile in highly selected refractory cases, particularly in non-Asian populations with advanced graft dysfunction. This distinction is clinically relevant because refractory anemia represents a narrower and more treatment-resistant phenotype with greater therapeutic uncertainty. Most available reports address post-transplant anemia as a broad entity [15,16,17,18,20,21], whereas evidence specifically focused on strictly ESA-hyporesponsive/refractory anemia remains scarce [19].

The aim of the present study is to evaluate the efficacy and safety of roxadustat in kidney transplant recipients with refractory anemia under real-world prospective follow-up conditions.

## 2. Materials and Methods

### 2.1. Study Design and Setting

We conducted a prospective single-center, non-randomized, single-arm cohort study involving active pharmacologic treatment in adult kidney transplant recipients followed in a tertiary nephrology-transplant program. The objective was to evaluate hematologic response, treatment dynamics, and short-term safety of roxadustat in a refractory anemia population under routine clinical practice conditions.

All included participants received roxadustat according to local protocol and regulatory guidance and were monitored at predefined time points. Because the primary purpose was to evaluate real-world effectiveness in a narrowly defined difficult to-treat group, each participant was assessed longitudinally with baseline and week-12 comparisons.

### 2.2. Eligibility Criteria

Inclusion Criteria were age ≥18 years, kidney transplant recipient under active nephrology follow-up and refractory anemia defined as hemoglobin ≤ 10 g/dL despite maximal ESA dosing during the previous 12 weeks. Before treatment initiation, patients underwent clinical and laboratory assessment to exclude other potentially correctable causes of poor erythropoietic response, including intercurrent illness, active bleeding, uncontrolled inflammatory or infectious conditions, and alternative hematologic causes as part of the daily clinical routine.

### 2.3. Treatment Protocol

All recipients had been treated with darbepoetin alfa 1.5 µg/kg/week subcutaneously for 12 weeks before being enrolled. Roxadustat was initiated two weeks after the last ESA dose; thus, ESA therapy was discontinued before roxadustat initiation. Administration was oral, three times per week. Initial dosing was individualized according to prior ESA exposure and product-label conversion recommendations [22]. Transfusions were not allowed and oral or intravenous iron was permitted during the follow-up by protocol if clinically indicated. No change in the immunosuppressive regimen was admitted during the study.

Treatment response and tolerability were reviewed every four weeks. Dose adaptation followed a predefined algorithm:

- Dose increase if hemoglobin remained <11 g/dL.
- Dose maintenance if hemoglobin was above target range and clinically stable.
- Dose reduction if hemoglobin exceeded 12.5 g/dL.
- Temporary suspension if hemoglobin exceeded 13 g/dL.

This algorithm was chosen to balance efficacy and safety, minimizing prolonged severe anemia while reducing risk associated with excessive hemoglobin rise.

### 2.4. Endpoints

The primary endpoint was the proportion of patients achieving hemoglobin > 11 g/dL after 12 weeks of roxadustat treatment. Secondary endpoints included changes from baseline to week 12 in: hemoglobin, ferritin, transferrin saturation index, estimated glomerular filtration rate (CKD-EPI formula), C-Reactive Protein (CRP).

Safety endpoint assessment consisted of recording adverse events during follow-up, including severity and relationship to treatment when clinically applicable.

### 2.5. Statistical Analysis

Continuous variables are reported as medians and interquartile ranges (IQRs), and categorical variables as counts and percentages. Given the small sample size and the expected non-normal distribution of clinical variables, paired comparisons between baseline and week 12 were performed using non-parametric methods. The primary endpoint was summarized as the proportion of patients achieving Hb > 11 g/dL at week 12. Secondary analyses evaluated within-patient changes in Hb, ferritin, transferrin saturation, CRP, and eGFR. All analyses were considered exploratory, and results were interpreted primarily on the basis of effect size, direction of change, and clinical coherence. Statistical analyses were performed using RStudio 2025.09 Cucumberleaf Sunflower (R Foundation for Statistical Computing, Vienna, Austria).

### 2.6. Ethics

All participants provided written informed consent before inclusion. The study followed the ethical principles of the Declaration of Helsinki and was approved by the institutional ethics committee (Comité Ético de Investigación con Medicamentos, Alicante General Hospital Department; code 2025-097).

## 3. Results

### 3.1. Participant Flow and Baseline Profile

A total of 22 kidney transplant recipients were initially enrolled. Two later withdrew consent, leaving 20 participants who completed the 12-week follow-up and were included in the efficacy and safety analysis. All endpoints were complete for all these patients.

The final cohort included 11 men and 9 women. The median age was 69.0 years (63.1–74.0), and the median post-transplant time was 62.5 months (12.0–201.5). Baseline kidney function was markedly reduced, with median estimated glomerular filtration rate of 16.5 mL/min/1.73 m^2^ (13.8–25.5), indicating advanced graft dysfunction in most cases (Table 1).

Maintenance immunosuppression consisted of sirolimus-based regimens: 8 recipients received sirolimus monotherapy, whereas 12 received sirolimus in combination with tacrolimus. No patients received Angiotensin-Converting Enzyme inhibitors or Angiotensin II Receptor Blockers when refractory anemia was diagnosed.

#### Primary Efficacy Endpoint

At week 12, 19 of 20 recipients (95%; exact binomial 95% CI, 75.1–99.9%) achieved hemoglobin > 11 g/dL, meeting the predefined primary endpoint. Hemoglobin increased from a median (IQR) of 9.1 g/dL (8.5–9.8) at baseline to 11.5 g/dL (11.2–12.1) at week 12 (Table 1). When assessed as an individual paired change, the median hemoglobin increase was +2.7 g/dL (IQR 1.7–3.4; Wilcoxon signed-rank *p* < 0.001).

Only one participant (case 18) did not reach the target threshold. Even in this non-responder, hemoglobin increased from 9.5 to 10.2 g/dL, suggesting partial hematologic response (Supplementary Table S1).

### 3.2. Dose Dynamics During Follow-Up

**Figure 1:**
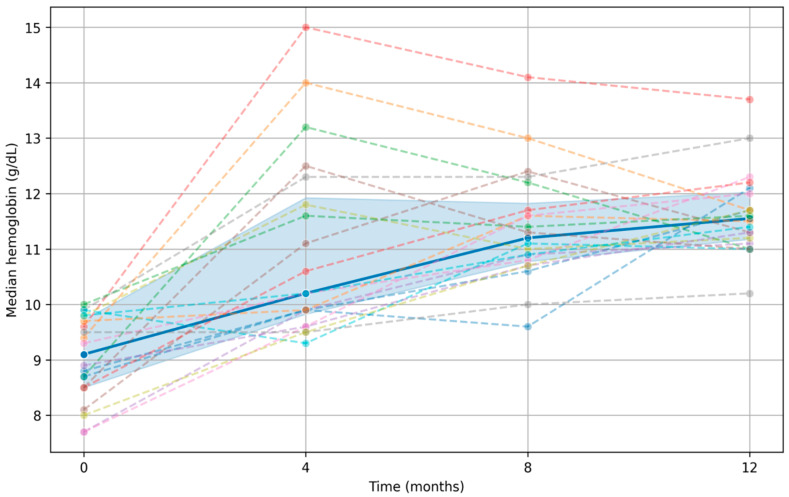
The solid blue line represents the median hemoglobin concentration at each time point, while the shaded area denotes the interquartile range (25th–75th percentiles). Dashed lines correspond to individual patient trajectories, connecting hemoglobin measurements across time.

Initial individual doses are reported in Supplementary Table S1. Dose adaptation reflected substantial individual variability in treatment response. During the 12-week period, eight patients (40%) required dose reduction, two (10%) required treatment suspension, six (30%) maintained the initial dose, and four (20%) required dose escalation. These patterns indicate that while most patients responded adequately, a substantial subgroup experienced a faster-than-expected increase in hemoglobin, requiring active downward adjustment to keep values in a safe range. Figure 1 shows the longitudinal hemoglobin trajectory of individual patients throughout the 12-week follow-up.

No participant required intravenous or oral iron supplementation during follow-up. Baseline ferritin was elevated in all cases (>170 µg/L), with a median of 329 µg/L. Despite apparently preserved iron stores, 9 recipients (45%) had transferrin saturation <20%, compatible with functional iron deficiency (Supplementary Table S1) [10].

Nine recipients also showed inflammatory activity, with CRP levels above the normal range (Supplementary Table S1). This laboratory profile supports the clinical relevance of studying roxadustat in this population, where inflammation-driven functional iron limitation is frequent [10,12].

Median individual changes from baseline to week 12 were −110.5 µg/L (IQR −159.8 to −36.5; *p* = 0.024) for ferritin, −3.5 percentage points (IQR −8.8 to 7.3; *p* = 0.390) for transferrin saturation, −0.15 mg/dL (IQR −0.75 to 0.05; *p* = 0.179) for CRP, and −1.0 mL/min/1.73 m^2^ (IQR −3.25 to 0.0; *p* = 0.106) for eGFR.

### 3.3. Safety Outcomes

No serious adverse events were recorded during the observation period. Two patients developed mild self-limited diarrhea episodes that did not require treatment discontinuation. No thromboembolic events or severe cardiovascular complications were observed in the short follow-up period. Overall, these findings suggest acceptable short-term tolerability under close clinical monitoring and proactive dose adaptation. However, the study was underpowered to assess uncommon or longer-term safety outcomes, particularly thrombotic and cardiovascular risk.

## 4. Discussion

This prospective cohort study in kidney transplant recipients with ESA-hyporesponsive anemia (8) showed a strong short-term hematologic signal after roxadustat initiation. The primary endpoint was achieved by 95% of participants, with clinically meaningful hemoglobin improvement over 12 weeks. However, because of the single-arm non-randomized design, these findings should be interpreted as associative rather than confirmatory, and causal attribution to roxadustat alone cannot be established.

These results are relevant for several reasons. First, the study population represented a difficult clinical scenario: older recipients, advanced graft dysfunction, previous maximal ESA exposure, and a high frequency of inflammatory and functional iron-restricted profiles. Second, response occurred without additional iron supplementation, supporting a potential role of roxadustat in improving iron utilization efficiency.

The current findings are consistent with previous reports suggesting the efficacy of roxadustat in posttransplant anemia [15,16,17,18,19,20,21], but the present cohort differs in its explicit focus on refractory anemia. Many published studies included broader post-transplant anemia populations, with heterogeneous prior ESA exposure and less strict resistance definitions [15,16,17,18,19,20,21]. Miki et al. reported significant hemoglobin improvement after 12 weeks in a Japanese retrospective cohort [15], but refractory status and inflammatory phenotype were not characterized with the same granularity. Hui Li et al. described similar long-term hemoglobin outcomes across treatment groups in early post-transplant anemia [16], suggesting roxadustat effectiveness, but in a substantially different clinical setting from late refractory disease. Naganuma et al. presented favorable early trends in a small case series [17], though very limited sample size and lower inflammatory burden constrained external validity. Kong et al. demonstrated superiority of roxadustat plus oral iron versus oral iron alone in a randomized framework [18], yet participants were not selected for ESA-refractory anemia. Finally, Jun Li et al. included a subgroup with ESA resistance [19], which offers the closest comparator. Their response rate among resistant cases was lower than in our cohort, 63% vs. 95%. Possible explanations include shorter treatment duration, lower baseline hemoglobin, and differences in patient phenotype, concomitant therapy, and ESA-resistance criteria [19]. Tang et al. provided a narrative review summarizing the available evidence [23], whereas Guenal et al. focused on early post-transplant anemia in a non-Asian population [20], and Ma et al. evaluated roxadustat in a randomized peri-transplant setting with delayed graft function as the primary endpoint. Therefore, our study addresses a narrower and clinically difficult-to-treat subgroup for which prospective data remain scarce.

The observed response pattern is biologically plausible. Refractory anemia in transplant recipients often reflects a combination of inadequate endogenous erythropoietin signaling [7] and disturbed iron trafficking in inflammatory conditions [10,12]. Roxadustat may act at both levels by enhancing endogenous EPO response and reducing hepcidin-mediated iron sequestration [11].

In our cohort, elevated ferritin coexisted with low transferrin saturation in many participants, a classic signature of functional iron restriction rather than absolute deficiency [10]. The modest but statistically significant decline in ferritin over 12 weeks, together with the hematologic response observed in the absence of iron supplementation, seems to be compatible with the increased mobilization and utilization of stored iron during active erythropoiesis. However, because transferrin saturation and CRP did not change significantly, this finding should be interpreted cautiously and should not be taken as proof of full correction of inflammation-related iron dysregulation. Rather, it provides an exploratory mechanistic signal supporting the biologic plausibility of roxadustat in this selected population. All our recipients were receiving sirolimus-based immunosuppression, a regimen that may itself contribute to anemia.

Another notable finding is the frequency of dose reduction for or temporary discontinuation of roxadustat (50% combined). This suggests that conversion from high ESA doses to roxadustat may produce brisk erythropoietic responses in selected patients, requiring careful individualized titration (Figure 1). Clinically, this reinforces the need for close early follow-up to prevent overshooting target hemoglobin ranges.

From a practical perspective, these findings support roxadustat as a potentially valuable option in kidney transplant recipients with persistent anemia despite standard ESA-based management. A response rate near 95% in a refractory cohort, even under observational conditions, is clinically meaningful. The study also highlights an operational message: efficacy and safety are strongly linked to active monitoring. The favorable safety profile may partly reflect structured 4-week reassessment and timely dose adjustments. Therefore, implementation in routine practice should include clear titration algorithms and early surveillance, especially during the first 2–3 months. In addition, the absence of supplemental iron requirements during follow-up may be relevant for patient convenience and resource utilization. Although this should not be generalized without controlled comparisons, it suggests that in selected inflammatory-functional deficiency phenotypes, roxadustat may reduce dependence on repeated iron administration.

In routine clinical practice, the use of roxadustat in kidney transplant recipients with refractory anemia should be integrated into a structured care pathway rather than applied ad hoc. Because these patients often present with graft dysfunction, complex immunosuppressive regimens, infection risk, and cardiovascular comorbidity, treatment initiation should be preceded by a standardized assessment to exclude reversible causes of poor ESA response, including occult bleeding, malnutrition, infection, and untreated deficiency states. Our findings also suggest that the early titration phase is particularly important, as a substantial proportion of patients required dose reduction or temporary suspension, indicating that conversion schemes should be considered initial guidance rather than fixed prescriptions and that closer hemoglobin monitoring may be warranted during the first weeks of therapy.

This study has several strengths: it prospectively evaluates a narrowly defined cohort with refractory anemia, rather than heterogeneous post-transplant anemia populations. The inclusion criteria required prior high ESA exposure, aligning with real-world definitions of resistance. Follow-up was protocolized, with regular reassessment and predefined dose-adjustment rules. Additionally, the cohort provides data from Caucasian recipients, complementing previously published evidence generally centered on Asian populations.

The study also has important limitations. The small sample size limits statistical precision and subgroup analyses, while the single-center design may reduce external generalizability. The follow-up duration was short, precluding robust assessment of long-term safety and graft outcomes. In addition, the absence of a comparator group does not allow exclusion of regression to the mean or delayed residual effects of prior ESA therapy as partial contributors to the observed hemoglobin improvement. Because all participants were receiving sirolimus-based immunosuppression, this characteristic should be regarded as a potential confounder and may limit extrapolation to transplant populations managed with different maintenance strategies.

Despite these constraints, the study provides focused prospective evidence in a clinically high-need subgroup where randomized data are still scarce.

Future studies should evaluate roxadustat in multicenter, adequately powered, controlled designs specifically targeting ESA-refractory post-transplant anemia. Key priorities include the assessment of long-term cardiovascular and thrombotic safety, as well as its effects on graft function trajectory and graft survival. In addition, patient-reported outcomes—such as fatigue, physical functioning, and quality of life—should be incorporated. Further work is also needed to explore biomarker-guided algorithms, including those based on inflammation, hepcidin, and iron utilization profiles, as well as to assess comparative cost-effectiveness versus optimized ESA plus iron strategies. Clarifying these areas will be essential before broad implementation as a standard. Despite these constraints, the study provides focused prospective evidence in a clinically high-need subgroup where randomized data are still scarce.

## 5. Conclusions

In this prospective single-center cohort of kidney transplant recipients with ESA-hyporesponsive anemia, roxadustat was associated with high rates of hemoglobin target attainment at 12 weeks and acceptable short-term tolerability under close monitoring. These findings are preliminary and hypothesis-generating, and they support further evaluation of roxadustat in selected refractory post-transplant anemia cases. Larger multicenter controlled studies with longer follow-up are needed to define comparative efficacy, long-term safety, and effects on graft and cardiovascular outcomes.

## Figures and Tables

**Table 1:** Demographic variables. Patient-level baseline and week-12 laboratory data.

Demographics and Baseline Characteristics				
Variable	**Baseline Median (IQR)**			
Age (years)	69.0 (63.0–74.0)			
Male sex, n (%)	11 (55%)			
Post-transplant (months)	62.5 (12.0 –201.5)			
Initial Roxadustat Dose (mg)	70 (70–100)			
Laboratory outcomes After 12 Weeks				
**Variable**	**Baseline Median (IQR)**	**Week 12 Median (IQR)**	**Δ Change Median (IQR)**	***p*** **-Value**
eGFR (mL/min/1.73 m^2^)	16.5 (13.8–25.5)	17.0 (13.0–24.0)	−1.0 (−3.25–0.0)	0.106
Ferritin (µg/L)	329 (257–541)	308 (192–477)	−110.5 (−159.8–−36.5)	0.024
Transferrin saturation (%)	26 (16–34)	21 (16–30)	−3.5 (−8.8–7.3)	0.390
CRP (mg/dL)	0.7 (0.3–2.1)	0.6 (0.2–1.2)	−0.15 (−0.75–0.05)	0.179
Hb (g/dL)	9.1 (8.5–9.8)	11.5 (11.1–12.1)	+2.7 (1.7–3.4)	<0.001

Values are median (IQR); Δ = week 12—baseline; Hb: hemoglobin; eGFR: glomerular filtration rate; CRP: C-reactive protein. *p*-values by Wilcoxon signed-rank test. N/A: not applicable.

## Data Availability

Data is unavailable due to privacy and ethical restrictions.

## Supplementary Materials

The following supporting information can be downloaded at https://www.mdpi.com/article/10.3390/life16050815/s1, Table S1: Demographic variables. Patient-level baseline and week-12 laboratory data.

## Abbreviations

Hb	hemoglobin
CKD	chronic kidney disease
ESAs	erythropoiesis stimulating agents
HIF-PHIs	hypoxia-inducible factor prolyl hydroxylase inhibitors
CRP	C-reactive protein
CI95%	confidence interval of 95
P25-P75	25th and 75th percentiles
