# Supplementary material for: Roxadustat in Kidney Transplant Recipients with ESA-Hyporesponsive Anemia: A Prospective Single-Center Cohort Study
Source: Life (Basel). 2026 May 13;16(5):815. doi: 10.3390/life16050815 (PMC13208854; doi:10.3390/life16050815)
Supplement: Supplementary file 1 [file life-16-00815-s001.zip › life-4270514-supplementary.pdf]

					Initial parameters					At 12 weeks of therapy				
n	Age(years)	Gender	post-tx	Initial Dose(mg)	Hb	eGFR	Ferritin	T Fe	CRP	Hb	GFR	Ferritin	T Fe	CRP
1	35	M	24	100	8.8	55	2047	17	7.7	11.7	51	661	15	2.6
2	75	F	260	200	9.4	14	170	48	1.2	11.7	14	661	43	0.6
3	71	F	24	70	8.7	11	587	32	0.4	11.0	9	1021	27	8.2
4	63	M	44	70	9.6	15	1400	16	2.4	13.7	20	539	31	0.9
5	74	F	240	70	7.7	11	347	32	0.1	11.1	10	200	21	0.9
6	73	M	240	70	8.1	13	186	18	2.8	11.3	9	155	12	1.9
7	72	M	144	70	9.3	16	297	12	1.2	12.3	16	102	20	0.5
8	64	F	10	50	9.9	35	1220	42	0.8	13.0	30	566	27	0.1
9	60	M	12	70	9.8	47	249	21	0.5	11.2	40	134	13	1.3
10	60	F	130	70	9.9	16	532	50	0.1	11.0	14	394	16	0.1
11	67	M	72	70	8.7	17	299	15	0.2	12.1	16	167	13	0.2
12	71	M	96	50	9.7	22	242	27	0.5	11.5	25	458	34	0.2
13	47	F	53	100	10.0	18	260	31	0.2	11.4	17	214	40	0.4
14	63	M	12	70	8.5	27	247	8	5.1	12.2	24	202	17	0.2
15	76	M	4	100	8.9	26	1247	48	1.8	11.3	15	379	17	2.4
16	66	F	4	100	8.5	14	567	38	0.5	11.0	25	535	21	0.5
17	77	M	204	150	7.7	22	310	13	2.5	12.0	21	162	16	2.3
18	74	F	260	100	9.5	8	414	24	0.3	10.2	6	317	18	0.3
19	75	M	6	100	8.0	24	404	15	0.2	11.7	25	298	31	0.1
20	63	F	192	100	9.8	9	302	28	2.4	11.4	9	264	29	1.1
Median	69	N/A	62.5	70	**9.1***	16.5	329	26	0.7	**11.5***	17	308	21	0.6
P25	63	N/A	12	70	8.5	13.8	257	16	0.3	11.1	13	192	16	0.2
P75	74	N/A	201	100	9.8	25.5	541	34	2.1	12.1	24	477	30	1.2

******p*<0.001**

Gender: male (M)/female(F); post-tx: (months post transplant); Hb: hemoglobin (g/dL); GFR glomerular filtration rate (ml/min/1.73m2); Ferritin (µg/L); T Fe: transferrin saturation (%); CRP: C reactive protein (mg/dL); N/A: not applicable

Table S1: Demographic variables. Patient-level baseline and week-12 laboratory data
